# SM08502-Mediated β-Catenin Repression Synergizes with Olaparib to Inhibit Tumor Progression

**DOI:** 10.1158/2767-9764.CRC-25-0267

**Published:** 2025-12-04

**Authors:** Bradley R. Corr, Elizabeth R. Woodruff, Tomomi M. Yamamoto, Kimberly R. Jordan, Thomas Danhorn, Carine Bossard, Lily L. Nguyen, Edward B. Chuong, Lars Wick, Alexandra Young, Shinya Kusumoto, Sandra Orsulic, Lisa Barroilhet, Benjamin G. Bitler

**Affiliations:** 1 Division of Gynecologic Oncology, University of Colorado Denver, Anschutz Medical Campus, Aurora, Colorado.; 2 Division of Reproductive Sciences, Department of Obstetrics and Gynecology, University of Colorado Denver, Anschutz Medical Campus, Aurora, Colorado.; 3 Department of Immunology, University of Colorado Denver, Anschutz Medical Campus, Aurora, Colorado.; 4 University of Colorado Cancer Center, Department of Biomedical Informatics, University of Colorado Denver, Anschutz Medical Campus, Aurora, Colorado.; 5 Biosplice, Inc., San Diego, California.; 6 Department of Molecular Cellular and Developmental Biology, BioFrontiers Institute, University of Colorado Boulder, Boulder, Colorado.; 7 Department of Biochemistry and Molecular Genetics, University of Colorado Anschutz Medical Campus, Aurora, Colorado.; 8 Caris Life Sciences, Phoenix, Arizona.; 9 Department of OB/GYN, UCLA David Geffen School of Medicine, Los Angeles, California.; 10 Jonsson Comprehensive Cancer Center, University of California Los Angeles, Los Angeles, California.; 11 Department of Veterans Affairs, Greater Los Angeles Healthcare System, Los Angeles, California.; 12 Division of Gynecologic Oncology, Department of Obstetrics and Gynecology, University of Wisconsin, Madison, Wisconsin.

## Abstract

PARP inhibitors (PARPi) have reshaped the clinical management paradigm of multiple cancers, but none more than homologous recombination–deficient high-grade serous carcinoma (HGSC) of tubo-ovarian origin. In patients with HGSC that harbor *BRCA1/2*-mutations, PARPi maintenance therapy after first-line chemotherapy has resulted in significantly prolonged progression-free and overall survival. However, PARPi resistance is a major clinical challenge, and subsequent therapeutic options for patients with resistant disease remain limited. Whereas mechanisms of PARPi therapy have been described, there have been few clinical studies to translate these strategies into overcoming resistance. Elevated WNT signaling and T-cell factor (TCF) transcriptional activity contribute to PARPi resistance; however, directly targeting WNT signaling is challenging due to on-target adverse events. We tested an indirect WNT inhibitor, a dual CDC-like kinase and dual-specificity tyrosine phosphorylation–regulated kinase inhibitor, SM08502 (cirtuvivint), in combination with PARPi in multiple resistant HGSC models. We determined TCF transcriptional activity and differential gene expression with splicing analysis and used multispectral IHC to interrogate the tumor microenvironment. In PARPi-resistant models, SM08502 inhibits WNT/TCF transcriptional activity, reduces cell viability, and induces DNA damage. In addition, using multiple immune-compromised and immune-intact *in vivo* models of PARPi-resistant disease, SM08502, in combination with olaparib, significantly reduces disease progression, remodels the tumor immune microenvironment, and extends survival. Specifically, tumors treated with the SM08502/olaparib combination exhibit reduced immune-suppressive PD-1 and PD-L1 expression. This study provides strong preclinical evidence that SM08502, in combination with PARPi, may be an effective strategy to overcome PARPi resistance.

**Significance:**

PARPi resistance is a major clinical challenge. Overcoming PARPi resistance will provide patients with therapeutic options. The study shows, in the context of resistant disease, the potential of targeting CDC-like kinase/dual-specificity tyrosine phosphorylation–regulated kinase alone and in combination with PARP inhibitors.

## Introduction

PARP inhibitors (PARPi) exploit deficiencies in DNA damage repair and have significantly altered the clinical care of multiple cancers. High-grade serous carcinomas of tubo-ovarian origin (HGSC) are frequently marked by elevated chromosomal instability, a hallmark of DNA repair deficiency, making them a particularly effective target for PARPi usage. Specifically, nearly 50% of HGSC tumors are proposed to be homologous recombination DNA repair–deficient (HRD; ref. 1), often through the mutation of HR pathway genes *BRCA1* and *BRCA2*. Notably, in patients with germline *BRCA1/2* mutations, the addition of olaparib, a PARPi, as a maintenance therapy following upfront chemotherapy led to a greater than 30-month progression-free survival benefit (2). Despite these paradigm-shifting findings, PARPi resistance and PARPi-related adverse events represent major clinical challenges.

Multiple mechanisms of PARPi resistance have been reported, including secondary *BRCA1/2* mutations, increased replication fork stabilization, epigenetic reprogramming, and differential signaling transduction (3–6). Notably, elevated WNT signaling attenuates PARPi efficacy in *BRCA2*-mutated HGSCs, and PARPi-resistant HGSCs have increased WNT/TCF target gene expression (6). These studies suggest that targeting WNT signaling could overcome PARPi resistance; however, the clinical use of WNT inhibitors has historically been challenging due to on-target adverse events (7).

WNT signaling is a complex signal transduction pathway primarily involved in developmental processes and immune cell regulation (8–10). WNT signaling functions through both β-catenin–dependent and –independent pathways. Normally, WNT ligands (e.g., WNT3A) bind to a membrane-bound frizzled receptor to sequester the β-catenin degradation complex composed of glycogen synthase kinase 3 β (GSK3β), APC, and Axin at the membrane. This leads to the accumulation of β-catenin in the cytosol, nuclear translocation, and its interaction with the T-cell factor (TCF) and lymphoid enhancer factor (LEF). Nuclear translocation of β-catenin leads to TCF-mediated transcriptional activation of target genes, including MYC or derepression of Groucho/TCF-mediated transcriptional targets. In multiple cancer types, the WNT/β-catenin pathway is aberrantly regulated via the loss of negative regulators (e.g., APC mutations) or β-catenin mutations that prevent its degradation. In HGSC tumors, despite the WNT/β-catenin pathway often being aberrantly regulated, components are rarely mutated, suggesting that the pathway is important for HGSC disease progression.

SM08502 (cirtuvivint) is a small-molecule pan-inhibitor of CDC-like kinase (CLK1-4) and dual-specificity tyrosine kinase (DYRK). This agent has been demonstrated to inhibit WNT pathway–related gene expression and protein expression through an alternative splicing mechanism in endometrial (bioRxiv 2023.04.04.535570), gastrointestinal, and hematologic malignancies (11, 12). SM08502 inhibits WNT signaling in colorectal, endometrial, and ovarian cancers (11, 13, 14) and is currently under investigation in early-phase clinical trials in solid and liquid tumor settings (13). WNT signaling is an attractive therapeutic target for multiple cancers; thus, we wanted to examine combination strategies with SM08502 in therapy-resistant HGSC.

In this report, we examined the use of a pan-CLK/DYRK inhibitor, SM08502, to indirectly inhibit WNT/TCF transcriptional targets as a strategy to overcome PARPi resistance in HGSC. Using several human and murine PARPi-resistant HGSC models, we observed that SM08502 significantly reduces TCF transcriptional activity, induces elevated DNA damage, and synergizes with olaparib to enhance antitumor activity in two independent immune-intact murine models as well as a patient-derived xenograft (PDX) immune-compromised model. These findings strongly support the clinical use of SM08502 against PARPi-resistant HGSC.

## Materials and Methods

### Cell lines, cell culture, and drugs

The parental PEO1 (TP53/BRCA2-mutated) cells are olaparib-sensitive (PEO1-S). PEO1 olaparib-resistant (PEO1-OR) cells were created by dose-escalation of olaparib (6, 14). UWB1.289 (*TP53/BRCA1*-mutated; ref. 15) cells were obtained from the ATCC and authenticated at the University of Arizona Genomics Core using short tandem repeat DNA profiling. UWB1.289 sensitive (UWB1.289-S) cells were treated via dose-escalating olaparib to generate UWB1.289 olaparib-resistant (UWB1.289-OR) cells. PEO1-OR cells were maintained in RPMI 1640 supplemented with 10% FBS from Phoenix Scientific (#PS-100) and 1% penicillin/streptomycin L-glutamine (Corning #30-009-CI). UWB1.289 cells were maintained in 50% RPMI 1640, 50% MEGM with its supplements (Lonza, #CC-3150), 3% FBS, and penicillin/streptomycin. Olaparib-resistant ID8 Tp53^−/−^ Brca2^−/−^ (ID8-OR) cells were maintained in DMEM (Corning #10-013-CV) supplemented with 4% FBS, 1% insulin/transferrin/selenium (Gibco #41400-045), and penicillin/streptomycin. Olaparib-resistant SO1 Tp53^−/−^ Brca1^−/−^, Hras, Myc (SO1-OR) cells were maintained in DMEM (Corning #10-013-CV) supplemented with 10% FBS and penicillin/streptomycin. Cells were maintained for a maximum of 20 passages in culture and tested regularly for *Mycoplasma* via LookOUT (Millipore-Sigma) or MycoStrip (InvivoGen) and were last confirmed negative on March 3, 2025. Drugs used include DMSO (1%, Thermo Fisher Scientific, #J66650.AP), olaparib (500 nmol/L–20 μmol/L, SelleckChem, #S1060), rucaparib (SelleckChem, #S4948), and talozoparib (SelleckChem, #S7048). SM08502 (a.k.a. cirtuvivint) was provided by Biosplice.

### Overexpression plasmids

Human GSK3β-myc was cloned from Tag5Amuc-GSK3g WT construct. Tag5Amyc-GSK3β WT was a gift from Mien-Chie Hung (Addgene plasmid #16260; http://n2t.net/addgene:16260; RRID: Addgene_16260). GSK3β -myc skipped exon (SE) mutant was created using all around PCR of Tag5Amuc-GSK3g WT as a template and using primers (GSK3β _del_R: tct​tga​gtggtg​aag​ttg​aag​agt​gc and GSK3β _del_F: gct​aat​act​gga​gac​cgt​gga​c). Both GSK3β -myc and SE mutant was then cloned into pCDH-blasticidin + adapter vector using primers (EcoRI_GSK3β _F: aaa​gaa​ttc​atg​tca​ggg​cgg​ccc and NotI_GSK3β _R: aaG​CGG​CCG​Cct​aca​gat​cct​ctt​cag​aga​tga​gtt​tct​gc). pCDH-blastidin + adapter vector was created by cutting out LaminA insert between BamHI and NotI and inserting additional XhoI, EcoRI, and restriction cutting site between BamHI and NotI. pCDHblast MCSNard OST-LMNA was a gift from Tom Misteli (Addgene plasmid #22661; http://n2t.net/addgene:22661; RRID: Addgene_22661).

### Retrovirus and lentivirus transduction

Retrovirus production and transduction were performed as described previously (16). Lentivirus was packaged using the ViraPower Kit from Life Technologies following the manufacturer’s instructions as described (17). *WNT3A* construct was described (6, 18). Cells transduced with virus encoding a puromycin resistance gene were selected in 1 μg/mL puromycin.

### Colony formation

HGSC cells were seeded and treated with increasing doses of olaparib, rucaparib, talazoparib, and SM08502 or in combination. Cell media and drugs were changed every 2 days for 12 days. Colonies were fixed (10% methanol/10% acetic acid) and stained with 0.4% crystal violet. Crystal violet was dissolved in fixative, and absorbance was measured at 570 nm and normalized to DMSO control. To determine the combination index (CI) of the combination treatment, CI values were determined via the CompuSyn software (19).

### TCF transcriptional reporter assay

TCF transcriptional activity was evaluated using a Luciferase Assay System (cat. E1501; Promega) and TOP-FLASH, FOP-FLASH plasmids (11, 20). Using FuGENE6 reagent (cat. #E2692; Promega), populations were transfected with TOP-FLASH or FOP-FLASH plasmid. M50 Super 8x TOPFlash and FOPFlash were provided by Randall Moon (cat. #12456/12457; Addgene; ref. 20). Cells were incubated for 24 hours and then moved to a 96-well plate and exposed to serial doses of SM08502 for 48 hours. Cells were then lysed and analyzed using the Luciferase Assay system with luminescence measured by a Promega GloMax.

To normalize this assay for transfection efficiency and cell count, FOP-FLASH luciferase activity and crystal violet were performed on each cell line. For FOP-FLASH transfected cells, luminescence was also quantified as a measure of transfection efficiency. For crystal violet, the cells were seeded, incubated, and treated in the same way as the cells in the Luciferase Assay System. Then, 48 hours after treatment, the cells were fixed (10% methanol, 10% acetic acid) and stained with 0.4% crystal violet. Crystal violet was dissolved in fixative, and absorbance was measured at 570 nm.

### Immunofluorescence

PEO1-S ,PEO1-OR, UWB1.289-S, and UWB1.289-OR cells were seeded into a 24-well plate containing glass coverslips at a density of 50,000 cells per well, 16 hours prior to drug treatment to allow for cell adherence. The EC_50_ concentrations of SM08502 and olaparib were determined using crystal violet assays. After the 16-hour incubation period, cells were treated under one of the following conditions: untreated, 0.1% DMSO (vehicle control), EC_50_ concentrations of SM08502, olaparib, or combination treatment with both SM08502 and olaparib. All treatments were conducted for 48 hours at 37°C. Following the 48-hour drug treatment, coverslips were processed for immunofluorescence (IF) microscopy. Coverslips were gently washed twice with PBS and fixed using 4% paraformaldehyde for 10 minutes at room temperature. The fixed cells were washed twice more with PBS and permeabilized in a PBS-Triton X-100 (0.1%) solution for 30 minutes at room temperature with gentle rocking. After permeabilization, coverslips were washed twice with PBS. Cells were blocked in PBS containing 5% BSA for 2 hours at room temperature with gentle rocking. Following blocking, coverslips were washed twice with PBS and incubated with the primary antibody solution. The γH2AX primary antibody (Millipore, cat. #05-636, RRID: AB_309864) was diluted 1:250 in PBS containing 2% BSA and applied to the cells. Coverslips were incubated overnight at 4°C with gentle rocking. After the overnight incubation, coverslips were washed three times with PBS, for 5 minutes each. Goat anti-Rabbit IgG (H + L) Highly Cross-Adsorbed Secondary Antibody, Alexa Fluor 546 (AF-546; Thermo Fisher Scientific, cat. #A-11035, RRID AB_2534093), was diluted 1:1,000 in PBS containing 2% BSA and applied to the cells for 2 hours at room temperature with gentle shaking, protected from light. Following secondary antibody staining, cells were washed three additional times with PBS, for 5 minutes each. Excess PBS was carefully aspirated, and coverslips were mounted onto glass slides using VECTASHIELD Antifade Mounting Medium with 4′,6-diamidino-2-phenylindole (DAPI; cat. #H-1200, Vectashield, Vector Laboratories Inc.). Mounted coverslips were sealed with clear nail polish and allowed to dry at room temperature. Coverslips were stored at 4°C, protected from light, until observation. IF images were captured using an Olympus FV-1000 confocal microscope. Sequential z-stack images were acquired using excitation lasers at wavelengths 405 nm (DAPI) and 543 nm (AF-546). For each sample, a minimum of three representative fields were imaged. Images were processed and analyzed using Fiji/Image J software (21).

### Immunoblotting

Cells were collected and washed once using cold PBS. Protein was extracted using RIPA buffer (50 mmol/L Tris-HCl pH 8, 1% NP-40, 0.5% sodium deoxycholate, 0.1% SDS, and 150 mmol/L NaCl) combined with protease and phosphatase inhibitor cocktail (Roche, cat. #05892791001). Protein concentration was measured using the bicinchoninic acid protein assay (Thermo Fisher Scientific, cat. #23227). A total of 25 μg of protein extract was separated on TGX gradient gels (Bio-Rad) and transferred onto a polyvinylidene difluoride membrane. The membrane was blocked by shaking for 30 minutes at room temperature in Licor blocking buffer (cat. #927). The membrane was incubated overnight, rocking at 4°C with primary antibodies diluted in Licor blocking buffer. Primary antibodies included β-catenin (Cell Signaling Technology, cat. #8480, RRID: AB_11127855), BRCA1 (Cell Signaling Technology, cat. #9010, RRID: AB_2228244), β-actin (Abcam, cat. #ab6276, RRID: AB_2223210), γH2Ax (Millipore, cat. #05-636, RRID: AB_309864), GAPDH (Cell Signaling Technology, cat. #97166, RRID: AB_2756824), GSK3β (Cell Signaling Technology, cat. #12456, RRID: AB_2636978), Myc-tag (Cell Signaling Technology, cat. #2276S, RRID: AB_331783), and histone H3 (Cell Signaling Technology, cat. #4499, RRID: AB_10544537). The next day, the membrane was washed three times with Tris-buffered saline with 0.1% Tween 20, and secondary goat anti‐rabbit (IRDye 680RD or IRDye 800CW, LI‐COR, cat. #92568071; RRID: AB_2721181 or cat. #926‐32211; RRID: AB_621842; 1:20,000) and goat anti‐mouse (IRDye 680RD or IRDye 800CW, LI‐COR, cat. #926‐68070; RRID: AB_10956588 or cat. #925‐32210; RRID: AB_2687825; 1:20,000) antibodies were applied for 1 hour at room temperature. Blots were visualized using the Licor Odyssey Imaging System and ImageStudio software (version 4).

### Cellular fractionation

Cells were seeded in six-well plates and treated with DMSO, olaparib (1 μmol/L), SM08502 (200 nmol/L), or combination of olaparib (1 μmol/L) and SM08502 (200 nmol/L) and incubated at 37°C for 24 hours. The cells were harvested using trypsin and washed with PBS. Cells were suspended into hypotonic buffer [10 mmol/L HEPES (pH7.9), 10 mmol/L KCl, 1.5 mmol/L MgCl2, 0.34 mol/L sucrose, 10% glycerol, 1 mmol/L DTT, 1x complete EDTA-free protease inhibitors, and 0.1% TritonX-100] and incubated on ice for 10 minutes. Next, part of the total cell lysate was collected before centrifugation and suspended into 5x Laemmli sample buffer. The remaining cell lysates were centrifuged at 1,300 × *g* for 5 minutes at 4°C. Pellets were washed with hypotonic buffer and centrifuged at 1,300 × *g* for 5 minutes, and pellets (nuclear fraction) were suspended in RIPA buffer (50 mmol/L Tris-HCl pH 8.0, 150 mmol/L NaCl, 1% TritonX-100, 0.5% sodium deoxycholate, 0.1% SDS, and 5 mmol/L EDTA), sonicated, and suspended in 5x Laemmli sample buffer. Supernatants (cytosol fraction) were further centrifuged at 13,000 × *g* for 5 minutes at 4°C and collected as cytosol fraction.

### *In vivo* experiments

All *in vivo* studies were approved by the Institutional Animal Care and Use Committee [protocol # 283 (PDX model) and 569 (syngeneic models)]. In all experiments, mice were treated daily via oral gavage with olaparib (50 mg/kg; LC Laboratories), SM08502 alone (25 mg/kg; Biosplice), combination olaparib + SM08502 (ola + SM), or vehicle (5% DMSO, 30% PEG300 in H_2_O). Body weights were collected twice weekly as a surrogate for general well-being, and blood chemistry was analyzed for drug toxicity in the syngeneic murine model, as described below. Primary measures of tumor burden were completed at necropsy.

### PDX

Six- to 8-week-old female NOD.Cg-Prkdc^scid^Il2rgt^m1Wjl^/SzJ mice (The Jackson Laboratory, strain #005557) were intraperitoneally injected with olaparib-resistant PDX-GTFB1016 cells (5 million cells per mouse; described in ref. 22). Collection and use of patient material was approved by the Colorado Medical Institutional Review Board (IRB #07-935) in accordance with the ethical guidelines described in the Helsinki Declaration, and patients provided informed written consent. Tumor cells were tagged with a GFP-luciferase reporter to enable tumor establishment validation via *in vivo* imaging (IVIS, PerkinElmer; refs. 6, 17). Specifically, 2 weeks after PDX-GTFB1016-OR cell injection, mice underwent IVIS scanning and were randomized into treatment groups (vehicle control, olaparib, SM08502, or ola + SM) based on total flux (photon/second). Starting the next day, mice received 28 daily treatments, followed by euthanasia and necropsy on the 29th day. At this time, ascites cells were collected for downstream analysis. The final tumor burden is reported in terms of ascites cell volume and solid tumor weight.

### Syngeneic murine model

Six- to 8-week-old female C57BL/6J mice (The Jackson Laboratory, strain #000664) were intraperitoneally injected with GFP-luciferase–tagged syngeneic olaparib-resistant ID8 Tp53^−/−^ Brca2^−/−^ cells (5 million cells per mouse; described in ref. 23). Mice were given 7 days to establish significant tumor burden, followed by IVIS scanning and randomization as described above. Starting the next day, mice received 28 daily treatments, followed by euthanasia and necropsy on the 29th day. At this time, omenta were collected for downstream analysis. The final tumor burden is reported in terms of omentum weight as well as secondary disseminated tumor quantity and total weight.

To enhance confidence in observed treatment effects, we utilized a unique olaparib-resistant syngeneic model, SO1-OR (generously provided by S. Orsulic, University of California Los Angeles). SO1-OR cells were generated through continuous exposure to high-dose olaparib. In these experiments, 6- to 8-week-old female C57BL/6J mice were intraperitoneally injected with cells (20,000 per mouse) and given a 24-hour tumor establishment period. In the first experiment, mice were given 21 daily treatments, followed by euthanasia and necropsy on the 22nd day. The final tumor burden is reported in terms of total tumor weight. For drug toxicity assessment, 50 to 100 μL blood was collected from mice via submandibular puncture on the last day of treatment, and complete blood count (CBC) as well as blood chemistry (aspartate aminotransferase and alanine transaminase) were analyzed on a Heska HemaTrue impedance analyzer through the Comparative Pathology Shared Resource at the University of Colorado Anschutz Medical Campus using HeskaView and proprietary software. Blood chemistry was only measured in samples with sufficient volume remaining after CBC. In the second experiment, we examined the benefit of treatment on overall survival (OS). In this study, mice received up to 28 daily treatments, depending on the number of days elapsed before reaching humane endpoint status.

### RNA sequencing

Ascites was collected from PDX-GTFB1016-OR tumor-bearing mice during necropsy and centrifuged at 1,000 RPM for 5 minutes. The supernatant was removed, and ascites cells were washed twice with sterile PBS. Cells were then immediately resuspended in RNAlater, incubated at 4°C for 24 hours, and then stored at −20°C. RNA was extracted and purified using RNeasy Plus Mini Kit (Qiagen, cat. #74136) as per the manufacturer’s instructions.

Sequencing libraries were prepared from the RNA harvested from ascites-associated cells from four mice per treatment group. According to the manufacturer’s protocols, ribosome depletion and library preparation were performed using the Qiagen FastSelect (cat. #334375) and KAPA BioSystems mRNA HyperPrep Kit (cat. #KK8581). Briefly, 500 ng of RNA was used as input, and KAPA BioSystems single-index adapters were added at a final concentration of 1.5 mmol/L. Purified, adapter-ligated library was amplified for a total of 11 cycles following the manufacturer’s protocol. The final libraries were pooled and sequenced on an Illumina NovaSeq X plus (University of Colorado Genomics Core; RRID: SCR_021984) as 150 bp paired-end reads.

RNA sequencing (RNA-seq) data were analyzed by the Bioinformatics and Biostatistics Shared Resource of the University of Colorado Cancer Center (RRID: SCR_021983) using the nf-core/rnasplicing Nextflow pipeline version 1.0.3 (24). The pipeline uses the STAR (RRID: SCR_004463) aligner to map sequence reads to the genome, in this case, human (GRCh38) and mouse (GRCm39) combined. The gene annotations used were from Ensembl release 104 (http://may2021.archive.ensembl.org/, RRID: SCR_002344). Gene expression was quantified using Salmon (RRID: SCR_017036) and converted to a table with the tximport R package (RRID: SCR_016752) in the pipeline. Ensembl IDs in the raw-counts table were converted to gene names, and the counts of genes with multiple IDs were aggregated. Counts were normalized to counts per million, and genes with a mean count <1 counts per million were excluded from differential gene expression analysis, which was performed using the limma R package (version 3.46.0; RRID: SCR_010943; ref. 25) with the voom function in R version 4.0.2 (https://www.R-project.org/; RRID: SCR_001905). Using the Reactome TCF-dependent signaling in response to WNT pathway, we generated a heatmap of expression of pathway genes via *Z*-score normalization and unsupervised clustering. *P* values were adjusted for multiple testing using the Benjamini–Hochberg method (26). Principal component analysis was performed with the prcomp function in R 4.0.2 (RRID: SCR_014676). Splicing changes were analyzed using rMATS (RRID: SCR_023485; ref. 27) and the DEXSeq package (RRID: SCR_012823; ref. 28) as part of the nf-core/rnasplicing pipeline. Differential splicing events between treatments detected in rMATS using both junction counts and exon body counts with a FDR of 0.05 or less were counted for each of the five categories (SE, retained intron, mutually exclusive exons, as well as alternative 5′ and 3′ splice sites). Pathway analyses were performed with the clusterProfiler R package (version 3.18.1; RRID: SCR_016884; ref. 29) using the msigdbr (version 7.5.1; RRID: SCR_022870) database.

### PCR/GSK3B sequencing

Cells were treated with either DMSO or combination of olaparib (1 μmol/L) and SM08502 (200 nmol/L) for 24 hours. Total RNA was purified using RNeasy Plus Mini kit (Qiagen, cat. #74136). A measure of 100 ng of total RNA, 10 μL of 2x Luna Universal One-Step reaction mix, 1 μL of 20x RT enzyme mix (New England Biolabs, E3005), 0.8 μL of 10 μmol/L of GSK3B_Univeral_R primer (CCA​AAC​GTG​ACC​AGT​GTT​GC), and 0.8 μL of 10 μmol/L of GSK3B_Universal_F primer (TCA​AAT​TAA​GGC​ACA​TCC​TTG​GAC) were mixed in 20 μL reaction, and reverse transcription followed by PCR for 40 cycles were done by following the manufacturer’s instructions. Whole RT-PCR reactions were loaded onto 2% agarose gel, and excised bands (∼460 bp and ∼360-390 bp) were purified using PureLink Quick Gel Extraction Kit (Invitrogen, cat. #K210012) and submitted to Sanger sequencing with GSK3B_Univeral_R primer (CCA​AAC​GTG​ACC​AGT​GTT​GC; Quintara Biosciences).

### Multispectral IHC

Multispectral IHC analyses were performed using Polaris Automated Quantitative Pathology Systems (Akoya Biosciences) as described (30). Murine tumor sections were sequentially stained with antibodies specific for γH2Ax (1:400, Opal 620, Abcam, cat. #ab2893, RRID: AB_303388), Pd-l1 (1:100, Opal 690, R and D Systems, cat. #AF1019, RRID: AB_354540), cleaved caspase 3 (1:100, Opal 540, Cell Signaling Technology, cat. #9661, RRID: AB_2341188), Cd3 (1:50, Opal 520, Cell Signaling Technology, cat. #99940, RRID: AB_2755035), Wt1 (1:400, Opal 650, Novus, cat. #NB110-60011B, RRID: AB_1849479), Pd-1 (1:150, Opal 570, Cell Signaling Technology, cat. #84651, RRID: AB_2800041), Ki67 (1:400, Opal 480, Thermo Fisher Scientific, cat. #RM-9106, RRID: AB_2341197), and F4/80 (1:50, Opal 780, Cell Signaling Technology, cat. #30325, RRID: AB_2798990). Five tumor regions of interest were selected from each slide and imaged using the 20x objective on the Polaris microscope (Akoya Biosystems). Image analysis was performed using inForm software version 2.3 (Akoya), including tissue segmentation to define cells within the tumor regions (tumor-associated cells), cell segmentation to define cellular borders, and phenotyping to assign each cell to a phenotypic category.

### Caris molecular data

Real-world OS information was obtained from insurance claims data for patients whose tumor samples underwent comprehensive molecular profiling at a Clinical Laboratory Improvement Amendments–certified laboratory (Caris Life Sciences). Caris Life Sciences approved the University of Colorado IRB (COMIRB# 25-119) for the use of the data and figure. OS was calculated from the time of initial date of diagnosis to death/last contact for patients (*N* = 1,407) with HGSC whose biopsies were collected from the primary tumor site (fallopian tube, ovary, or tubo-ovarian) and were treated with a PARPi (olaparib, niraparib, rucaparib, or talazoparib). Patients were stratified based on the median expression of *TCF7L1* (median = 5.59 transcripts per million) and *TCF7L2* (median = 93.37 transcripts per million).

### Statistics

Graphs and statistical analysis were completed in GraphPad Prism (version 10, RRID: SCR_002798). Data are presented as the mean ± SEM. An unpaired Student *t* test was used for statistical comparison between the control and treatment groups. Dose–response curves were calculated using nonlinear regression via the log(inhibitor) versus response equation. A one-way ANOVA was used to determine variance among multiple gestational groups, with a Benjamini–Hochberg Multiple Comparison *post hoc* correction to determine significance between individual groups. A *P* value or adjusted (adj.) *P* value of < 0.05 was considered significant.

## Results

### Hyperactivation of WNT signaling promotes PARPi resistance

Patients with germline *BRCA1* and *BRCA2* mutations are prescribed PARPis; however, the development of therapy resistance is common and represents a major clinical challenge. Using PEO1 (*BRCA2*-mutated cells) and UWB1.289 (*BRCA1*-mutated cells), we developed isogenic pairs of PARPi-sensitive (-S), -resistant (-OR), and WNT3A overexpression (-WNT3A; Supplementary Fig. S1A) lines. Dose escalation of olaparib was used to develop the resistant cell line. We previously determined PEO1-OR cells do not exhibit reversion mutations of *BRCA2* (6). In UWB1.289-OR cells, using an N-terminal antibody, we did not observe expression of a reverted protein, suggesting an alternative mechanism of olaparib resistance (Supplementary Fig. S1B).

**Figure 1.**
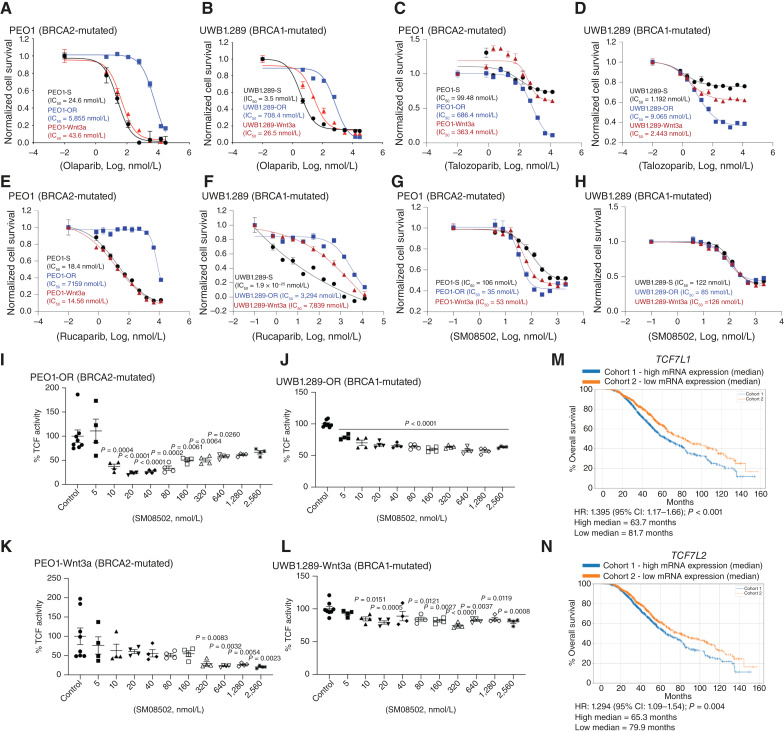
WNT signaling and TCF activity promote PARPi resistance and are sensitive to SM08502. **A,** PARPi-sensitive (-S) PEO1 (BRCA2-mutated) and (**B**) UWB1.289 (BRCA1-mutated) cell lines were transduced with WNT3A (-Wnt3a) or developed to be olaparib-resistant (-OR). Cells were treated with increasing doses of olaparib, and cell survival was measured via colony formation. **C,** PEO1 (-S, -Wnt3a, and -OR) and (**D**) UWB1.289 (-S, -Wnt3a, and -OR) cells treated with increasing doses of talozoparib. **E,** PEO1 (-S, -Wnt3a, and -OR) and (**F**) UWB1.289 (-S, -Wnt3a, and -OR) cells were treated with increasing doses of rucaparib. **G,** PEO1 (-S, -Wnt3a, and -OR) and (**H**) UWB1.289 (-S, -Wnt3a, and -OR) cells were treated with increasing doses of a novel pan-CLK/DYRK inhibitor—SM08502. **I,** PEO1-OR and (**J**) UWB1.289-OR cells were treated with increasing doses of SM08502, and TCF transcriptional activity was measured via TOP/FOP-FLASH assay. **K,** PEO1-Wnt3a and (**L**) UWB1.289-Wnt3a cells were treated with increasing doses of SM08502, and TCF transcriptional activity was measured via TOP/FOP-FLASH assay. **M,** Patients with HGSC treated with PARPis stratified based on the median of *TCF7L1* mRNA expression. **N,** Same as (**M**) but with median *TCF7L2* mRNA expression. Error bars, SEM. Statistical test, nonlinear regression, ANOVA with Tukey multiple comparison test, Kaplan–Meier with log-rank. IC_50_ concentration is shown as an inset for each graph. CI, confidence interval.

We confirmed that both the resistant and WNT3A-overexpressing cell lines had increased IC_50_ concentrations for olaparib compared with the sensitive lines (Fig. 1A and B). To ensure this was not specific to olaparib, we also confirmed the sensitivity to two other PARPis, talozoparib and rucaparib. In both the PEO1 and UWB cell lines, we observed that the olaparib-resistant lines were cross-resistant to the different PARPis. In the PEO1 and UWB1.289 cell lines, the overexpression of WNT3A resulted in an increased IC_50_ value of talozoparib (Fig. 1C and D). With respect to rucaparib, WNT3A overexpression only increased the IC_50_ value in the UWB1.289 cell line, not the PEO1 cell line (Fig. 1E and F). These studies demonstrate that overexpression of WNT3A generally leads to a decreased PARPi inhibitory response.

Targeting WNT signaling is an approach to overcome PARPi resistance, for example, by using a novel inhibitor of pan-CLK/DYRK to indirectly inhibit WNT signaling. SM08502 is an orally available pan-CLK/DYRK inhibitor that is effective at inhibiting multiple tumor types and has been demonstrated to inhibit WNT-mediated TCF activity (bioRxiv 2023.04.04.535570; refs. 11, 12). In the isogenic-paired cell lines, SM08502 effectively inhibited tumor cell viability at nanomolar ranges in the sensitive, resistant, and WNT3A-overexpressing cell lines (Fig. 1G and H).

Upon WNT ligand activation, β-catenin accumulates in the cytosol and translocates into the nucleus to promote transcription of TCF target genes. We have previously observed that PARPi-resistant HGSC cells have a significant increase in TCF activity and target genes (6). In both PARPi-resistant and WNT-overexpressing HGSC cells, we next wanted to confirm that SM08502 inhibited TCF transcriptional activity. Using the TCF reporter system TOP/FOP-FLASH (31), we observed that SM08502 significantly reduced TCF transcriptional activity across various doses (Fig. 1I–L; raw data in Supplementary Table S2), which is consistent with the loss of cell viability. These data highlight that WNT activation can drive therapy resistance and that SM08502 is highly effective at inhibiting cell viability and reducing TCF transcriptional activity.

In the context of therapy-resistant HGSC, elevated WNT signaling and TCF activity, compared with reversion mutations of HR genes, are more challenging to delineate due to multiple inputs that lead to TCF activity. Thus, to determine the impact of differential TCF activity on HGSC patient prognosis, we assessed the mRNA expression of *TCF7L1* and *TCF7L2* in a cohort of 1,407 primary patient tumors with HGSC treated with PARPis. The patients were identified through a Clinical Laboratory Improvement Amendments–certified laboratory (Caris Life Science). Using median expression of either TCF7L1 or 2 to analyze the data, we observed that elevated expression of these transcription factors led to a significantly worse OS—TCF7L1 (*N* = 640; HR: 1.395; *P* < 0.001) and TCF7L2 (*N* = 640; HR: 1.294; *P* = 0.004; Fig. 1M and N). Together, these data indicate that the increased expression of the TCF transcription factors predicts worse survival.

### Combination of SM08502 and olaparib is synergistic in olaparib-resistant cells

**Figure 2.**
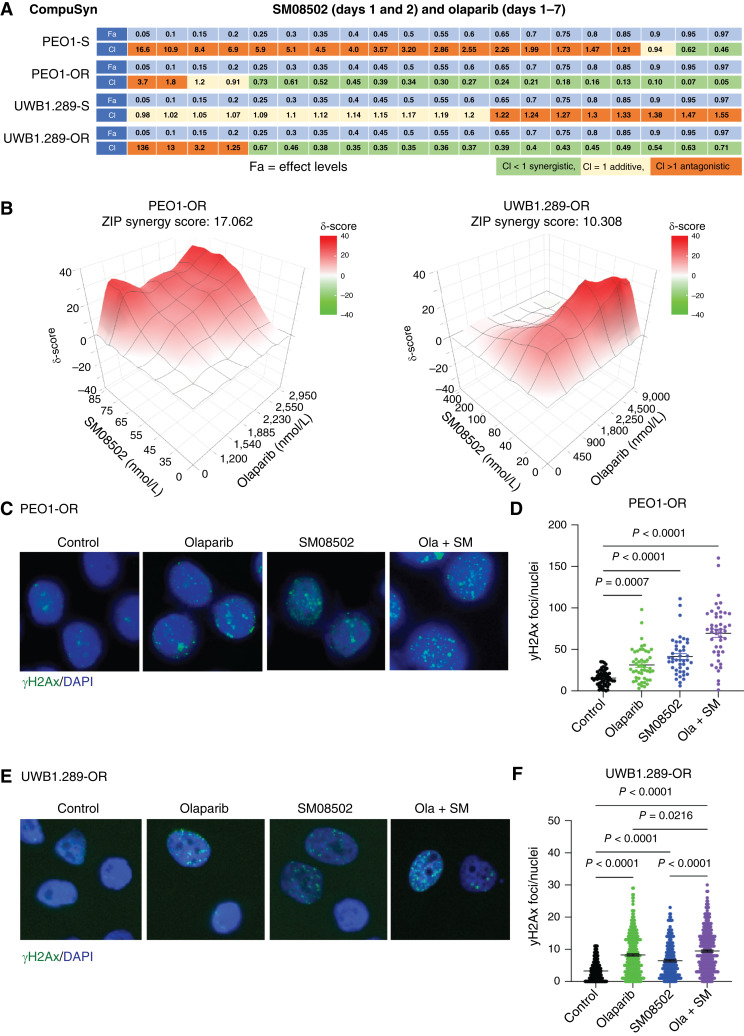
SM08502 and olaparib synergize to inhibit cell viability and promote DNA damage in olaparib-resistant cells. **A,** Indicated cell lines were treated for 7 days with increasing doses of both SM08502 (only treated on days 1 and 2) and olaparib (days 1 through 7). Cell viability was assessed via colony formation. The CI was calculated at all the dose combinations via CompuSyn software using the Chou–Talalay method for drug combination. **B,** Similar to (**A**), PEO1-OR and UWB1.289-OR cells were treated with several doses of olaparib and SM08502. The data were graphed via Large Language Models curve fitting, and synergy was calculated via the ZIP method. ZIP synergy score >10 was considered synergistic. **C,** PEO1-OR cells were treated with olaparib, SM08502, or in combination (ola + SM). Cells were fixed and used for IF against γH2ax (green) and nuclei (DAPI, blue). **D,** γH2ax were counted in the PEO1-OR cells. **E,** Same as (**B**) but used UWB1.289-OR cells. **F,** γH2ax were counted in the UWB1.289-OR cells. Error bars, SEM. Statistical test, one way ANOVA with Tukey multiple comparison correction.

As elevated WNT signaling promotes PARPi resistance, we next wanted to assess the potential additive or synergistic effects of SM08502 and PARPi in two isogeneic matched cell lines (PEO1/PEO1-OR and UWB1.289/UWB1.289-OR). The CI was calculated using both the Chou–Talalay and ZIP methods (19). First, the CI was determined in the PEO1-S and UWB1.289-S cell lines (Supplementary Fig. S2A). In UWB1.289 cells, the combination SM08502 and olaparib was predominantly additive at lower doses and antagonistic at higher doses (Fig. 2A; Supplementary Fig. S2B). In PEO1-S cells, the combination was predominantly antagonistic, with synergy only observed at higher doses (Fig. 2A). In contrast, the CI for PEO1-OR and UWB1.289-OR demonstrated synergy at most dose effect levels. The antagonism observed at the extremes of the concentration gradient reflects a reported limitation to the Chou–Talalay method (32); thus, we confirmed synergy using the ZIP method. In the olaparib-resistant models, the combination was considered significant with ZIP synergy scores of 17.1 and 10.3 in PEO1-OR and UWB1.289-OR, respectively (Fig. 2B). Taken together, in the PARPi-resistant context, the combination of olaparib and SM08502 was deemed to be synergistic at most dose ranges, potentially highlighting the dependence of the WNT pathway in these cells.

PARPis primarily function by exploiting DNA damage repair deficiencies, leading to an accumulation of DNA damage selectively in DNA repair–deficient cells. We previously observed that both in olaparib-resistant cells and -sensitive cells with overactivation of WNT, there was an increase in DNA damage repair as measured via γH2Ax resolution (6). In PARPi-resistant cells, we next assessed γH2Ax foci formation following the combination of olaparib and SM08502. In both PEO1-OR and UWB1.289-OR cells, the combination led to an increase in γH2Ax foci generation compared with the control and single agents alone (Fig. 2C–F), suggesting that the synergy observed depends on the impact of inducing DNA damage.

### SM08502 alone and in combination with olaparib reduces HGSC PDX tumor progression

**Figure 3.**
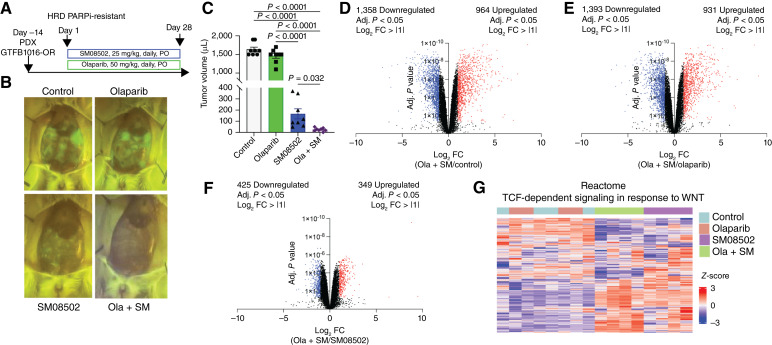
SM08502 reduces HGSC tumor growth and attenuates TCF transcriptional activity in olaparib-resistant tumors. **A,** GFP-expressing PDX-GTFB1016-OR tumors in NSG mice treated with olaparib, SM08502, or in combination (ola + SM). PO, orally. **B,** Representative GFP images on day 28. **C,** Tumor volume at the time of necropsy. **D,** Volcano plot of genes in ola + SM compared with control. Red dots = adj. *P* < 0.05, FC > 1. Blue dots = adj. *P* < 0.05, FC < −1. **E,** Volcano plot of genes in ola + SM compared with olaparib. Red dots = adj. *P* < 0.05, FC > 1. Blue dots = adj. *P* < 0.05, FC < −1. **F,** Volcano plot of genes in ola + SM compared with SM08502 alone. Red dots = adj. *P* < 0.05, FC > 1. Blue dots = adj. *P* < 0.05, FC < −1. **G,** Heatmap of Reactome TCF-dependent signaling in response to WNT pathway (also see Supplementary Table S3). Error bars, SEM. Statistical test, one way ANOVA with Tukey multiple comparison correction (**C**), unpaired *t* test with Benjamini–Hochberg correction for adj. *P* value (**D–F**).

Next, we wanted to assess the potential of combining SM08502 with olaparib to inhibit tumor progression in an established BRCA2-mutated olaparib-resistant PDX model, PDX-GTFB1016-OR, in immunodeficient mice (22, 33). PDX-GTFB1016-OR tumor-bearing mice were administered olaparib and/or SM08502 via oral gavage daily for 28 days (Fig. 3A). Based on body weight, olaparib and SM08502 were tolerated, with the combination showing a 5% loss in body weight over the course of the study (Supplementary Fig. S3A). At the end of the study, we confirmed that the tumors were resistant to olaparib. In contrast, SM08502 showed a significant antitumor effect compared with control. This effect was potentiated by the addition of olaparib, which led to a significant reduction in tumor burden compared with control and single agents alone (Fig. 3B and C).

To further define the mechanism of action in the tumor cells of olaparib- and SM08502-treated mice, we performed transcriptomic analysis via RNA-seq of the PDX-GTFB1016-OR tumors. Consistent with the olaparib-resistant context, we observed via principal component analysis that the transcriptomic profiles for the control- and olaparib-treated tumors were highly similar to one another (Supplementary Fig. S3B). However, the SM08502 and combination (ola + SM) tumors displayed distinct but different transcriptomic profiles, highlighting that the inhibitory mechanisms involved with olaparib and SM08502 are unique. Based on differential gene expression (adj. *P* < 0.05, Log_2_ fold change > |1|), the combination compared with control resulted in 2,322 differentially expressed genes (1,358 downregulated and 964 upregulated; Fig. 3D). Similarly, tumors treated with the combination compared with olaparib resulted in 2,324 differentially expressed genes (1,393 downregulated and 931 upregulated; Fig. 3E). In contrast, the combination treatment compared with SM08502 alone resulted in 774 differentially expressed genes (425 downregulated and 349 upregulated; Fig. 3F). Assessing genes associated with a TCF response following WNT activation, there was a reprogramming of target genes in the combination group compared with the control, olaparib, or SM08502 alone (Fig. 3G; Supplementary Table S2), suggesting a unique transcriptional regulation with the combination.

### SM08502 leads to differential splicing and reduced nuclear β-catenin localization

**Figure 4.**
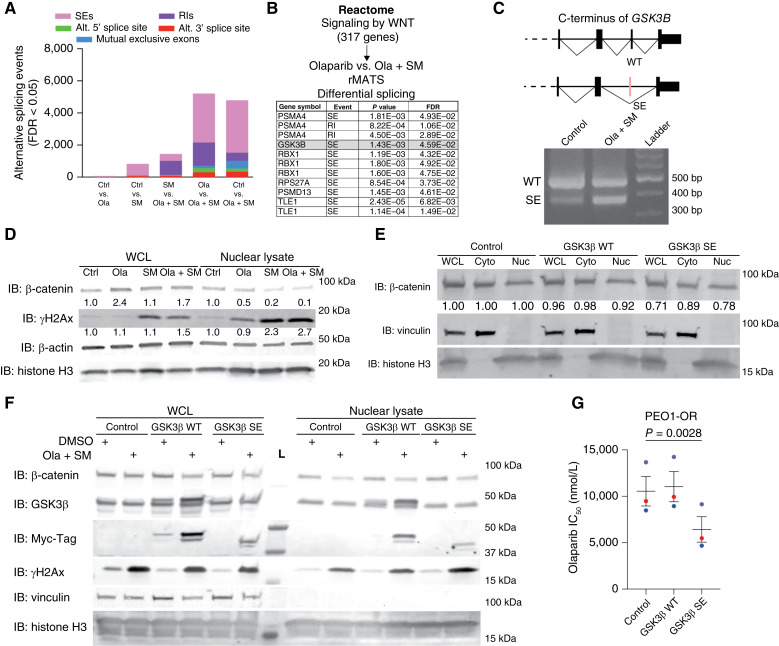
Transcriptomic and splicing analysis of PDX-GTFB1016-OR tumors reveals an elevated IFNα response and differential splicing of GSK3β. **A,** Splicing analysis of RNA-seq data. **B,** Assessing differential splicing of genes upregulated following WNT activation (Reactome gene set) between olaparib alone vs. the combination of olaparib and SM08502 (ola + SM). **C,** Differential splicing schematic of GSK3β and confirmation of differential GSK3β splicing via PCR of cDNA from control-treated or ola + SM–treated PEO1-OR cells. **D,** PEO1-OR cells treated with control, olaparib, SM08502, or ola + SM. Whole cell protein and nuclear lysates were isolated and used for immunoblotting against β-catenin and γH2Ax. Loading controls, β-actin and histone H3. Densitometry values calculated relative to histone H3. **E,** PEO1-OR cells were transfected with control, WT GSK3β, and GSKβ SE. Whole cell lysate (WCL) protein, cytoplasmic, and nuclear lysates were isolated and used for immunoblotting against β-catenin. Loading controls, vinculin and histone H3. Densitometry values calculated relative to vinculin and histone H3. Cyto, cytoplasmic; Nuc, nuclear. **F,** PEO1-OR cells were transfected with control, WT GSK3β, and GSKβ SE and treated with olaparib and SM08502 (ola + SM) for 72 hours. Nuclear protein extracts were isolated and used for immunoblotting. **G,** PEO1-OR cells were transfected with control, WT GSK3β, and SE GSKβ and treated with increasing doses of olaparib. IC_50_ values shown are calculated via a variable slope sigmoidal dose–response curve. The color dots represent independent biological replicates. Loading control, histone H3. Error bars, SEM. Statistical test, paired *t* test. Ctrl, control; IB, immunoblot; RI, retained intron.

CLK family members (CLK1, CLK2, CLK3, and CLK4) are directly involved in alternative splicing regulation by phosphorylating serine/arginine-rich splicing factors (34, 35), and inhibition of CLK/DYRK can inhibit WNT signaling via altering splicing of WNT pathway genes, including TCF/LEF transcription factors (11). Using the RNA-seq data, we performed a splicing analysis to understand the extent of differential splicing events following SM08502 and olaparib treatment. In comparing against splicing events in the control-treated tumors, we observed that treatment with both combination SM08502 and olaparib, as well as SM08502 alone, led to increases in alternative splicing events, specifically exon skipping (Fig. 4A). Compared with olaparib-treated tumors, tumors treated with SM08502 with or without olaparib showed a significant increase (adj. *P* < 0.05) in splicing events, specifically SEs and retained introns (Fig. 4A). Examining the connection between WNT signaling and differential splicing, we observed that ola + SM treatment led to a differential splice variant of several WNT/TCF-related genes, including GSK3β, produced by a SE within the 3′ end of GSK3β transcript (Fig. 4B and C). Note, we did not observe differential splicing of *BRCA1* or *BRCA2*. The alternatively spliced GSK3β is predicted to lack a whole exon that contains MAPK p38-directed inhibitory phosphorylation site, serine 389 (36). The phosphorylation of S389-GSK3β is proposed to depend on the induction of DNA double-strand breaks, consistent with the mechanism olaparib (36). The pS389-GSK3β leads to elevated activity, which can phosphorylate β-catenin, thereby promoting its degradation. Consistently, SM08502 alone and in combination with olaparib led to a reduction in nuclear β-catenin (control = 1.0 vs. SM08502 = 0.2 vs. ola + SM = 0.1 relative units; Fig. 4D; Supplementary Fig. S4). Similarly to the IF findings (Fig. 2), we observed olaparib-induced limited γH2Ax expression but noted an increase in γH2Ax following treatment with SM08502 alone or in combination with olaparib, indicative of greater DNA damage. We next developed an overexpression construct of wildtype (WT) GSK3β and GSK3β with SE to determine whethrt the expression of the GSK3β SE was sufficient to increase the sensitivity of olaparib. We observed that overexpression of GSK3β SE alone without treatment could reduce β-catenin levels by approximately 25% (Fig. 4E). In PEO1-OR cells following treatment with ola + SM, the expression of endogenous and exogenous GSK3β, both WT and SE, was elevated. Furthermore, β-catenin levels were reduced in the whole cell lysate, and the GSK3β SE expression led to elevated γH2Ax (Fig. 4F). In three independent experiments, compared with control, GSK3β SE expression led to a significant decrease in the IC_50_ value of olaparib in PEO1-OR cells (Fig. 4G). These data highlight a potential mechanism of SM08502-mediated alternative splicing modulation of WNT/ β-catenin signaling and its impact on DNA damage accumulation.

### SM08502 and olaparib are effective in immune-intact HGSC olaparib-resistant models

**Figure 5.**
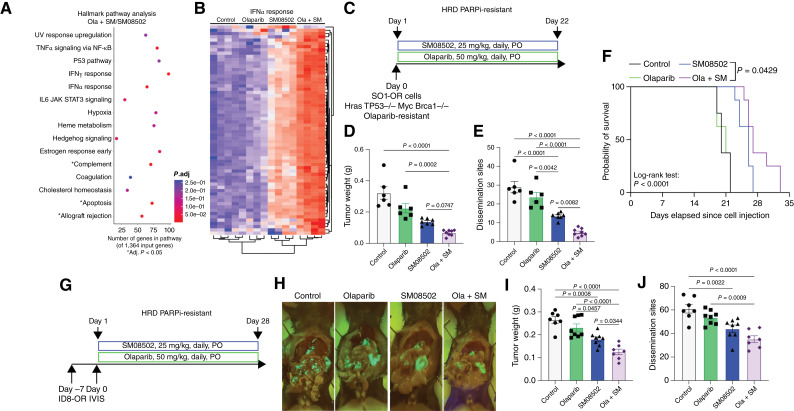
SM08502 overcomes PARPi resistance to reduce tumor burden and extend OS. **A,** Overrepresentation plot of Hallmark pathway enrichment of RNA-seq data from PDX-GTFB1016-OR. **B,** Heatmap of IFNα response genes of RNA-seq data from PDX-GTFB1016-OR. **C,** SO1 (Tp53^−/−^, Brca1^−/−^, Hras, and Myc)-OR murine cells were implanted via intraperitoneal injection, and mice were treated with olaparib, SM08502, or in combination (ola + SM). **D,** Day 22 tumor weight and (**E**) day 22 number of disseminated tumors. **F,** Survival of tumor-bearing control and treated mice. **G,** ID8 (Tp53^−/−^, Brca2^−/−^) OR murine cells were implanted via intraperitoneal injection, and mice were treated with olaparib, SM08502, or ola + SM. PO, orally. **H,** Representative day 28 necropsy images. GFP signal = tumor burden. **I,** Day 28 tumor weight and (**J**) day 28 number of disseminated tumors. Error bars, SEM. Statistical test, one way ANOVA with multiple comparison correction (**D**, **E**, **I**, and **J**) and Kaplan–Meier with log-rank test (**F**).

Targeting WNT signaling and alternative splicing are linked to elevated immune regulation in several cancer types (10, 37). Using the PDX-GTFB1016-OR transcriptomic data, we performed Hallmark pathway analysis on the tumors treated with ola + SM compared with SM08502 alone. Of the six significantly (adj. *P* < 0.05, Log_2_, fold change > |1|) enriched pathways, five were immune-related, including the IFNα and IFNγ responses, allograft rejection, and complement pathways (Fig. 5A). In the IFNα response pathway, there is a striking upregulation of most pathway-related genes in both SM08502 alone and in combination with olaparib (Fig. 5B). Whereas these data were generated in an immune-compromised setting, the robust enrichment of immune-related pathways led us to hypothesize that the combination of olaparib and SM08502 may have differential efficacy in an immune intact model.

To determine the impact of treatment in an immune intact setting, we next examined two syngeneic HGSC models that are HRD- and PARPi-resistant SO1-OR (Tp53^−/−^, Brca1^−/−^, Hras, and Myc; ref. 38) and ID8-OR (GFP, Luciferase, Tp53^−/−^, and Brca2^−/−^; ref. 23). In the aggressive SO1-OR model, mice were treated daily with vehicle (control), olaparib, SM08502, or combination (ola + SM) for 22 days (Fig. 5C). We confirmed that the SO1-OR model was olaparib-resistant (Fig. 5D). Compared with control and single agents alone, the ola + SM combination significantly reduced tumor burden and tumor dissemination (Fig. 5D and E). The observed tumor inhibition with ola + SM led to a significant survival benefit compared with SM08502 alone (*P* = 0.0429; Fig. 5F). No significant differences between any treatment groups were observed in body weight change or CBC and blood chemistry (aspartate aminotransferase and alanine transaminase) measures after 22 days of treatment, revealing no overt signs of drug toxicity (Supplementary Fig. S5B and S5C).

In the ID8-OR model, using a similar study design (Fig. 5G), we observed that the ola + SM combination significantly reduced omental tumor weight compared with the control and single agents alone (Fig. 5H and I). Also, SM08502 alone and combined with olaparib effectively reduced tumor dissemination compared with olaparib alone and control (Fig. 5J). In all animal studies, no overt toxicity was observed based on measurements of mouse body weight change (Supplementary Fig. S5A–S5C). We also performed a comprehensive hematologic evaluation and observed that several cell types were significantly decreased in the treated mice compared with the control mice. Notably, we observed the expected modest decreases in white blood cells and monocytes in treated mice but no significant variation amongst treatment arms (Supplementary Fig. S5C). These studies suggest the combination treatment is tolerated with limited adverse effects. The toxicity observed is consistent with preliminary phase I data (13). Of note, the observed clinical toxicities, diarrhea, and nausea, are difficult to assess in preclinical models. Taken together, in two independent *in vivo* models, the use of SM08502 alone and in combination with olaparib effectively inhibited tumor progression.

### Immune microenvironment of olaparib- and SM08502-treated tumors

**Figure 6.**
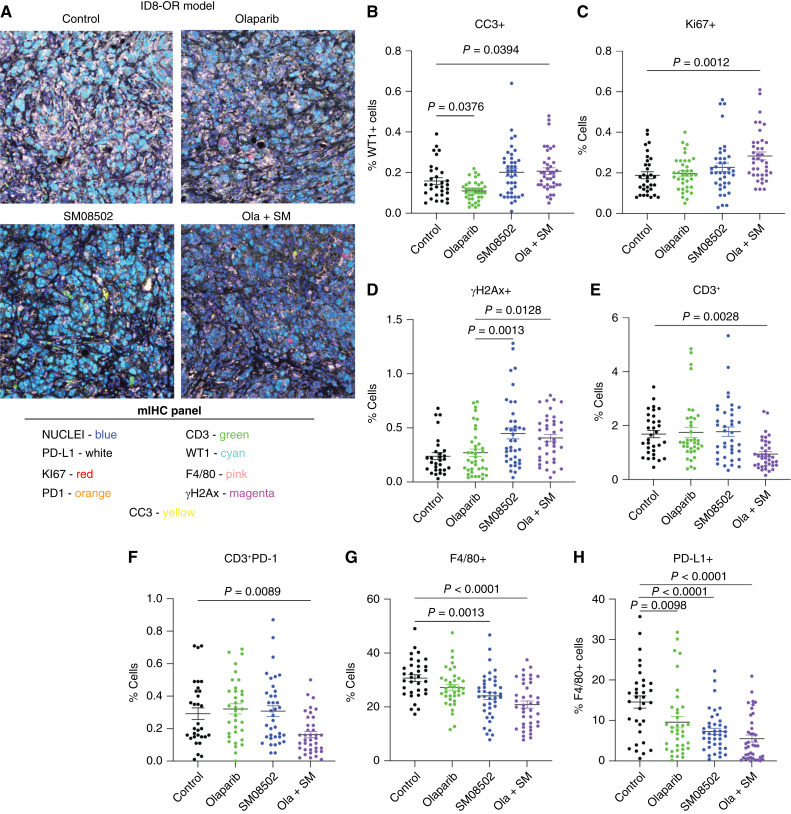
SM08502 and olaparib remodel the immune TME. **A,** ID8-OR tumors from mice treated with control, olaparib, SM08502, or in combination (ola + SM) were used for multispectral IHC. Five tumor regions were selected from each mouse (*n* = 5). **B,** Assessment of apoptosis via cleaved caspase 3 (CC3)-positive tumor (WT1+) cells. **C,** Assessment of proliferation Ki67-positive cells. **D,** Assessment of DNA damage via γH2Ax. **E,** CD3^+^ (T cells) and (**F**) PD-1+CD3^+^ (PD-1–expressing T cells). **G,** Tumor-associated macrophages (F4/80+). **H,** PD-L1+ expression in F4/80+ cells. Outlier analysis via ROUT (Q = 1%) was conducted on all graphs. Error bars, SEM. Statistical test, one-way ANOVA with Tukey multiple comparison correction.

In multiple immune-intact murine models of HGSC, the combination of SM08502 and olaparib significantly reduced tumor progression. We next used multispectral IHC to interrogate the impact of the combination on the omental tumor-immune microenvironment (TME; Fig. 6A). Compared with control-treated tumors, SM08052 in combination with olaparib led to increased expression of the apoptotic marker, cleaved caspase 3, in tumor cells (Fig. 6B). Compared with control, we detected that the combination therapy led to a modest, significant 0.1% increase in Ki67-positive cells within the TME (0.18% vs. 0.28%; *P* = 0.0012), potentially reflecting an outgrowth of a resistant population (Fig. 6C). Consistent with the *in vitro* evaluation, compared with olaparib alone, there was a significant increase in γH2Ax in the SM08502 alone and ola + SM groups (Fig. 6D). We detected a significant depletion of total CD3^+^ cells and PD-1–expressing T cells (CD3^+^PD-1+) in the combination therapy compared with control and single agents (Fig. 6E and F). However, the functional status of these T cells is unknown. Macrophages are proposed to be directly involved in HGSC disease progression and therapy resistance (39, 40). We observed that F4/80+ (macrophages) cells were depleted following both SM08502 alone and ola + SM compared with control tumors (Fig. 6G). Moreover, immune-suppressive macrophages (F4/80+, PD-L1+) were significantly reduced in all treatments and nearly depleted in the combination-treated tumors (Fig. 6H). Similarly to the T cells, the functional status of these macrophages is unknown. Together, the combination therapy uniquely remodels the immune TME.

## Discussion

Overcoming or delaying PARPi resistance remains a significant clinical challenge. Numerous studies have identified mechanisms of PARPi resistance; however, few have been translated to the clinic because of high levels of toxicity (41) and limited lead pharmacologic inhibitors (6). Using multiple models of PARPi-resistant HGSC, this study demonstrates the use of a pan-CLK/DYRK inhibitor, SM08502, to significantly inhibit WNT/TCF signaling, reduce cancer cell viability, and modulate alternative splicing. The combination of SM08502 and olaparib was synergistic at reducing cell viability, regulating alternative splicing, and remodeling the immune TME. Moreover, we found that SM08502 alone could drive an antitumor response in PARPi-resistant tumor models. These data strongly support using SM08502 with PARPi to treat patients with PARPi-resistant disease.

In colorectal cancer models, SM08502 significantly inhibits WNT signaling (11), and we extended these findings to therapy-resistant HGSC models. In addition, we observed a novel mechanism in a differentially spliced GSK3β. Also, a recent study by Chehade and colleagues (42) dissected the impact of *BRCA1/2* mutations on WNT signaling. The study reports that in isogeneic HGSC cell lines, both β-catenin (canonical WNT signaling) and actin cytoskeleton dynamics (noncanonical WNT signaling) are differentially regulated in the context of BRCA1/2 mutations, suggesting a targetable vulnerability in tumors that will also likely be receiving PARPi therapy(42). The transcriptional counterpart to TCF, LEF, was recently demonstrated to promote PARPi resistance (43). Furthermore, the data from more than 1,400 patients provides compelling evidence that TCF activity contributes to worse prognosis (Fig. 1M and N). the limitations of the data include the use of insurance data to provide date ranges and the inability to match pre- and post-PARPi transcriptomic information. Whereas directly targeting the WNT signaling pathway has been problematic because of toxicities, the indirect approach via TCF targeting mediated by SM08502 was tolerated in the tested models.

In addition, WNT/TCF signaling is crucial for normal T-cell development and the generation of memory T cells; thus, monitoring adaptive immunity capacity will be important as clinical trials progress. Whereas we noted that, compared with the control, the combination of SM08502 with olaparib decreased tumor-associated T cells (CD3^+^ cells) and PD-1–expressing T cells (CD3^+^PD-1+ cells), SM08502 or olaparib alone did not affect T-cell infiltration into the TME. Whereas cytotoxic and memory T cells are essential to drive antitumor immunity, immune-suppressive T regulatory cells can aid disease progression. Also, whereas we did observe shifts in tumor-associated myeloid composition (i.e., reduction in F480-positive cells), the CBC analysis highlighted systemic decrease in circulating monocytes, suggesting a nontumor-specific mechanism of monocyte reduction. In the future, it will be critical to ascertain the effect of SM08502 on T-cell and myeloid cell function and differentiation. Together, DYRK/CLK inhibition as an indirect approach to target the WNT signaling pathway is an attractive approach that circumvents the limitations of prior WNT-targeting strategies.

SM08502 inhibits the activity of CLK/DYRK, and CLKs are directly involved in the phosphorylation of serine/arginine-rich splicing factors within the spliceosome, leading to the dysregulation of splicing (44, 45). Whereas our study focused on the differential splicing of WNT/TCF-related genes, the data generated will allow for further interrogation of novel splicing events. Targeting alternative splicing in cancer cells is a promising approach for several reasons, including alternative splicing of DNA damage repair effectors to improve the efficacy of DNA-damaging agents, generation of neoantigens that may enhance immune surveillance of the tumors, and inhibiting cancer such as acute myeloid leukemia (AML) that are dependent on splicing machinery for disease progression. Xu and colleagues (46) reported a molecular glue, indisulam, which inhibits spliceosome machinery (DCAF15 and RBM39), resulting in an enrichment of alternatively spliced DNA repair genes and increased sensitivity to PARPis. Sun and colleagues (47) found that knockdown of a splicing factor, heterogeneous nuclear ribonucleoprotein A1, in the ID8 syngeneic HGSC murine model led to the production of neoantigens and increased infiltration of CD8^+^ T cells. Note, the ID8 model in that study had intact p53, whereas our studies used the ID8 model lacking p53 or BRCA2, generated to be olaparib-resistant (23, 48). Lastly, AML and myelodysplastic syndrome (MDS) are often defined by atypical splicing of known AML oncogenic drivers, including *FLT3* and *NOTCH2* (49, 50), suggesting these tumors may be acutely sensitive to the targeting of the splicing machinery. Together, alternative splicing is an attractive therapeutic target that exploits several aspects of cancer biology.

Despite a relatively low risk, the gynecologic oncology community has taken close surveillance of the use of PARPis due to the known increased risk of MDS and AML (51, 52). SM08502 (aka cirtuvivint) is hypothesized to be a therapeutic approach to hematologic malignancies and is in an early phase clinical trial in patients with patients relapsed/refractory AML/MDS (NCT06484062). Thus, combining SM08502 with PARPis may provide additional benefits by targeting early, abnormal hematopoietic stem cells, the precursor for MDS/AML.

The mechanisms proposed in this study translate into the potential therapeutic approach for a combination of CLK/DYRK and PARP inhibition in HGSC. SM08502, in combination with olaparib, inhibits WNT/TCF activity, reduces cell viability, and induces DNA damage, indicating an effective treatment strategy in HRD HGSC. Notably, a phase I clinical trial (NCT06856499) is currently open to patients with BRCA/HRD platinum-resistant HGSC to evaluate the safety profile of olaparib in combination with SM08502 (cirtuvivint).

## Supplementary Material

Table S1 Counts per million for RNA-seq

Table S2 Raw TOP/FOP-FLASH data from Figure 1.

Table S3 Heatmap data

Figure S1 WNT3A overexpression

Figure S2 Colony Formation and Crystal Violet

Figure S3 In vivo body weight and PCA plot

Figure S4 Fractionation immunoblot

Figure S5 Complete Blood Cell Counts

## Data Availability

The data are available upon request and will be made available in the relevant repository when services are restored. Caris Life Sciences molecular data used in this study can be accessed through an agreement and an approved IRB protocol. Requests can be initiated via https://www.carislifesciences.com/about/contact/. The transcript count table for the RNA-seq has been provided in Supplementary Table S1 and is available via Gene Expression Omnibus (GSE299344).
