# Supplementary material for: SM08502-Mediated β-Catenin Repression Synergizes with Olaparib to Inhibit Tumor Progression
Source: Cancer Res Commun. 2025 Dec 4;5(12):2112–26. doi: 10.1158/2767-9764.CRC-25-0267 (PMC12676110; doi:10.1158/2767-9764.CRC-25-0267)
Supplement: Figure S1 — WNT3A overexpression [file crc-25-0267_figure_s1_suppsf1.docx]

**
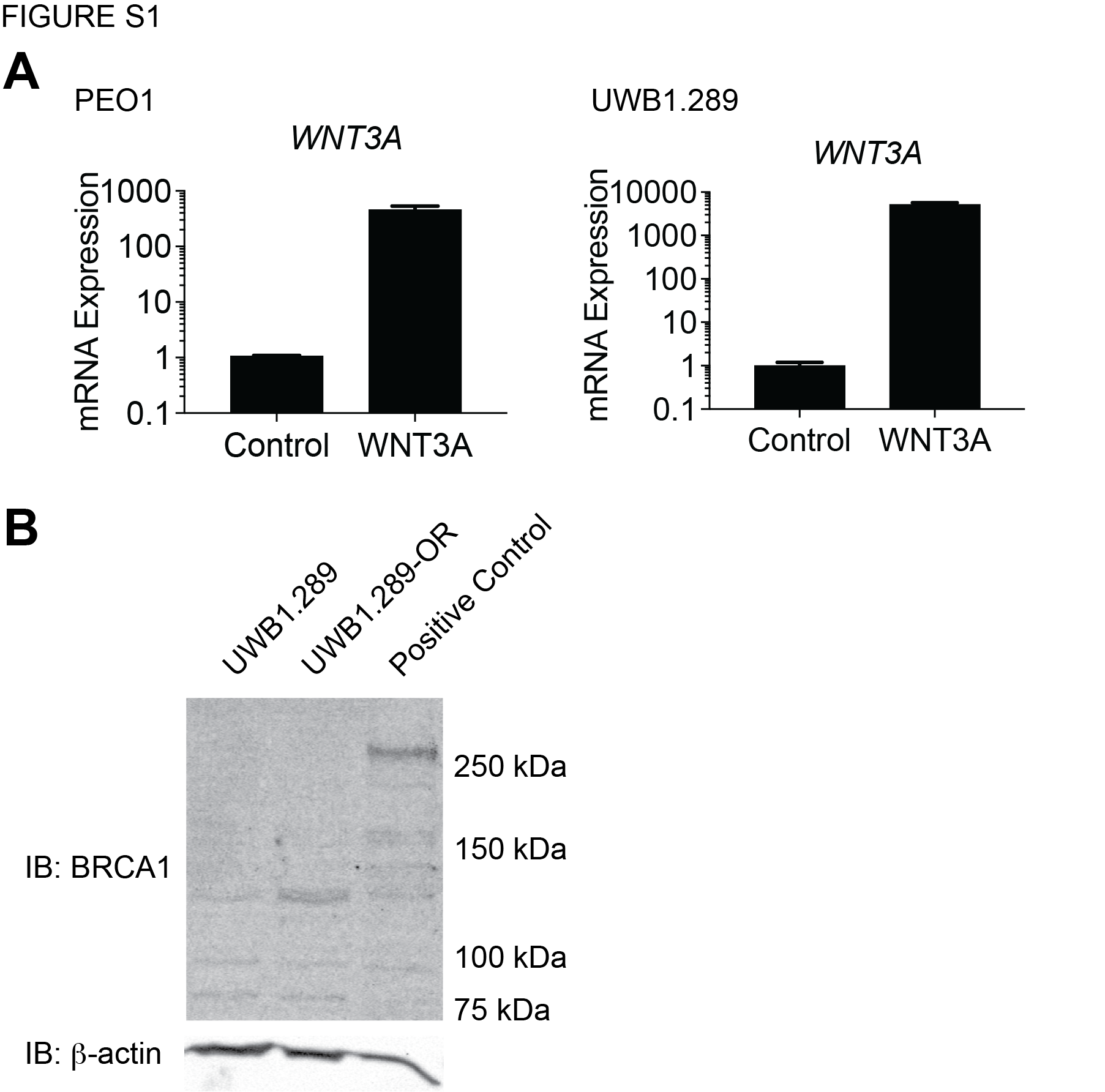
**

**Figure S1. WNT3A overexpression in homologous recombination deficient (HRD) high-grade serous carcinoma cell lines. A)** PEO1 and UWB1.289 olaparib sensitive cells were transduced with an empty vector (Control) or WNT3A overexpression construct. WNT3A mRNA expression was confirmed to be overexpressed via qPCR. Internal control, 18S. **B)** Protein isolated from UWB1.289 and UWB1.289-olaparib resistant (OR) cells was blotted for BRCA1. Loading control, β-actin. Error bars, SEM
