# Supplementary material for: SM08502-Mediated β-Catenin Repression Synergizes with Olaparib to Inhibit Tumor Progression
Source: Cancer Res Commun. 2025 Dec 4;5(12):2112–26. doi: 10.1158/2767-9764.CRC-25-0267 (PMC12676110; doi:10.1158/2767-9764.CRC-25-0267)
Supplement: Figure S2 — Colony Formation and Crystal Violet [file crc-25-0267_figure_s2_suppsf2.docx]

**
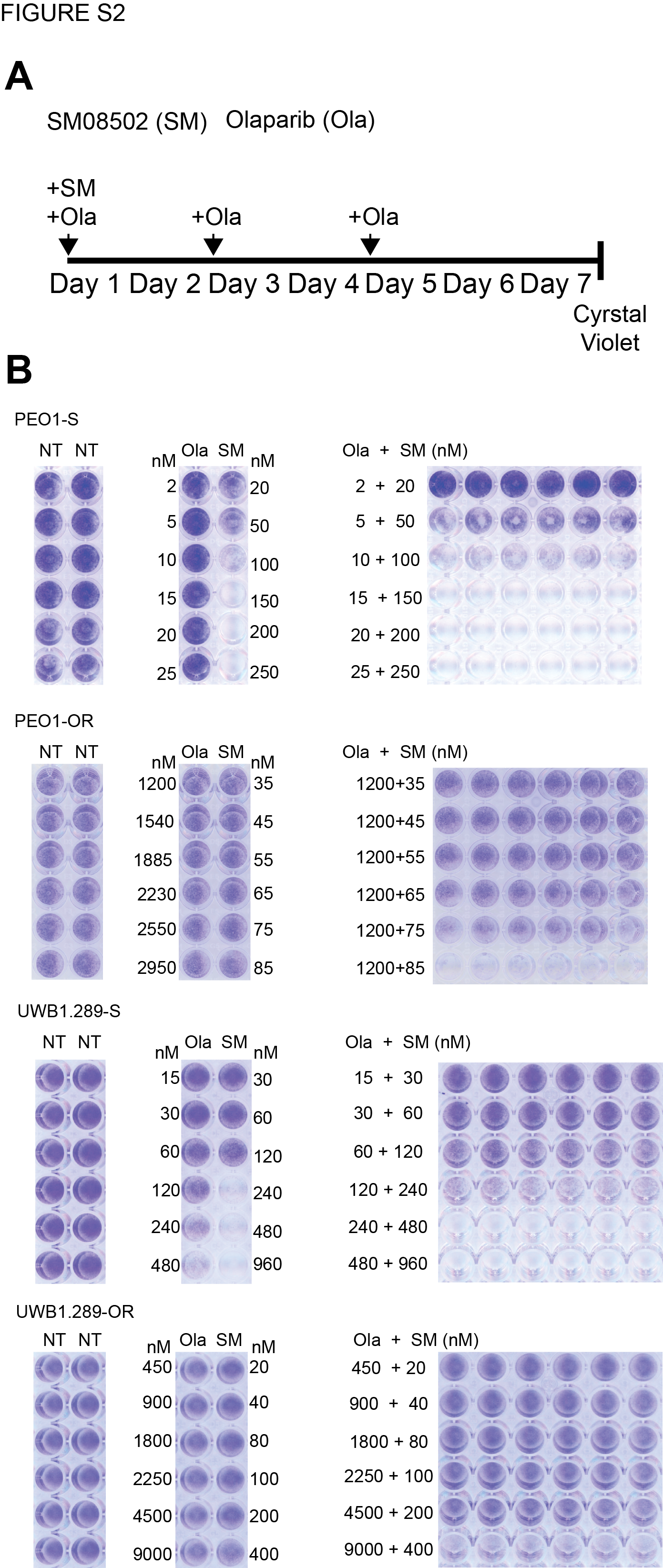
**

**Figure S2. Olaparib and SM08502 synergy assays. A)** Assay design. **B)** Representative images of colony formation assays assessing different doses of olaparib and SM08502. All doses are represented as nanomolar (nM).
