# Supplementary material for: SM08502-Mediated β-Catenin Repression Synergizes with Olaparib to Inhibit Tumor Progression
Source: Cancer Res Commun. 2025 Dec 4;5(12):2112–26. doi: 10.1158/2767-9764.CRC-25-0267 (PMC12676110; doi:10.1158/2767-9764.CRC-25-0267)
Supplement: Figure S3 — In vivo body weight and PCA plot [file crc-25-0267_figure_s3_suppsf3.docx]

**
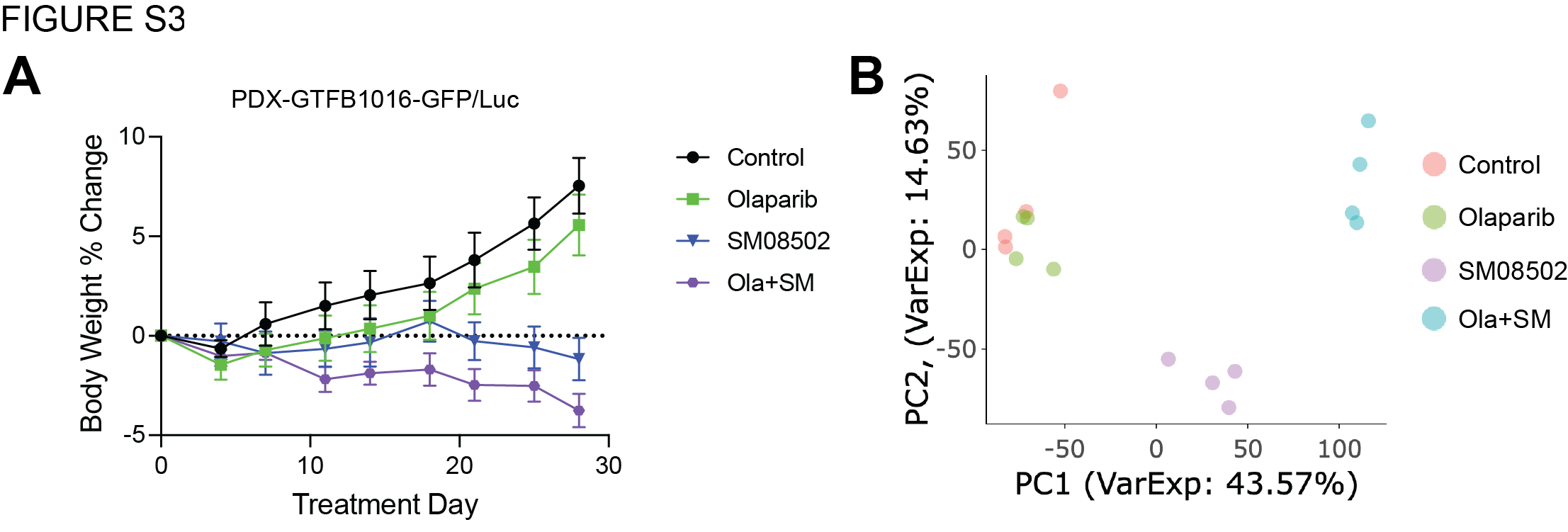
**

**Figure S3. Toxicity assessment in patient-derived xenograft models of olaparib resistant high-grade serous carcinoma. A)** PDX-GTFB1016-GFP-Luc tumor cells were orthotopically implanted and treated with control, olaparib (50 mg/kg, daily, oral gavage), SM08502 (25 mg/kg, daily, oral gavage), or in combination. Body weights are shown over time as percent change. **B)** Principal component analysis (PC) of RNA-seq data from PDX-GTFB1016-GFP-Luc treated tumors. Error bars, SEM.
