# Supplementary material for: SM08502-Mediated β-Catenin Repression Synergizes with Olaparib to Inhibit Tumor Progression
Source: Cancer Res Commun. 2025 Dec 4;5(12):2112–26. doi: 10.1158/2767-9764.CRC-25-0267 (PMC12676110; doi:10.1158/2767-9764.CRC-25-0267)
Supplement: Figure S4 — Fractionation immunoblot [file crc-25-0267_figure_s4_suppsf4.docx]

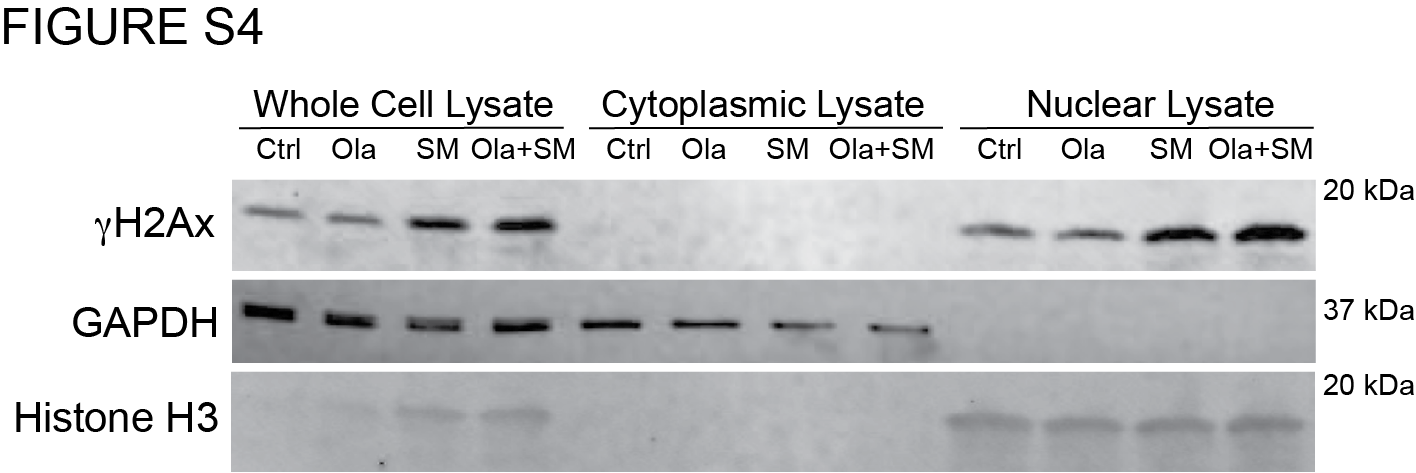


**Figure S4. Cellular Fractionation Controls. A)** PEO1-OR cells treated with control, olaparib (ola, 1 μM), SM08502 (SM, 200 nM), or in combination for 72 hrs. Cells were used for cytoplasmic and nuclear protein fractionation. Protein was blotted for gH2Ax (nuclear), GAPDH (cytoplasmic), and Histone H3 (nuclear).
