# Supplementary material for: SM08502-Mediated β-Catenin Repression Synergizes with Olaparib to Inhibit Tumor Progression
Source: Cancer Res Commun. 2025 Dec 4;5(12):2112–26. doi: 10.1158/2767-9764.CRC-25-0267 (PMC12676110; doi:10.1158/2767-9764.CRC-25-0267)
Supplement: Figure S5 — Complete Blood Cell Counts [file crc-25-0267_figure_s5_suppsf5.docx]

**
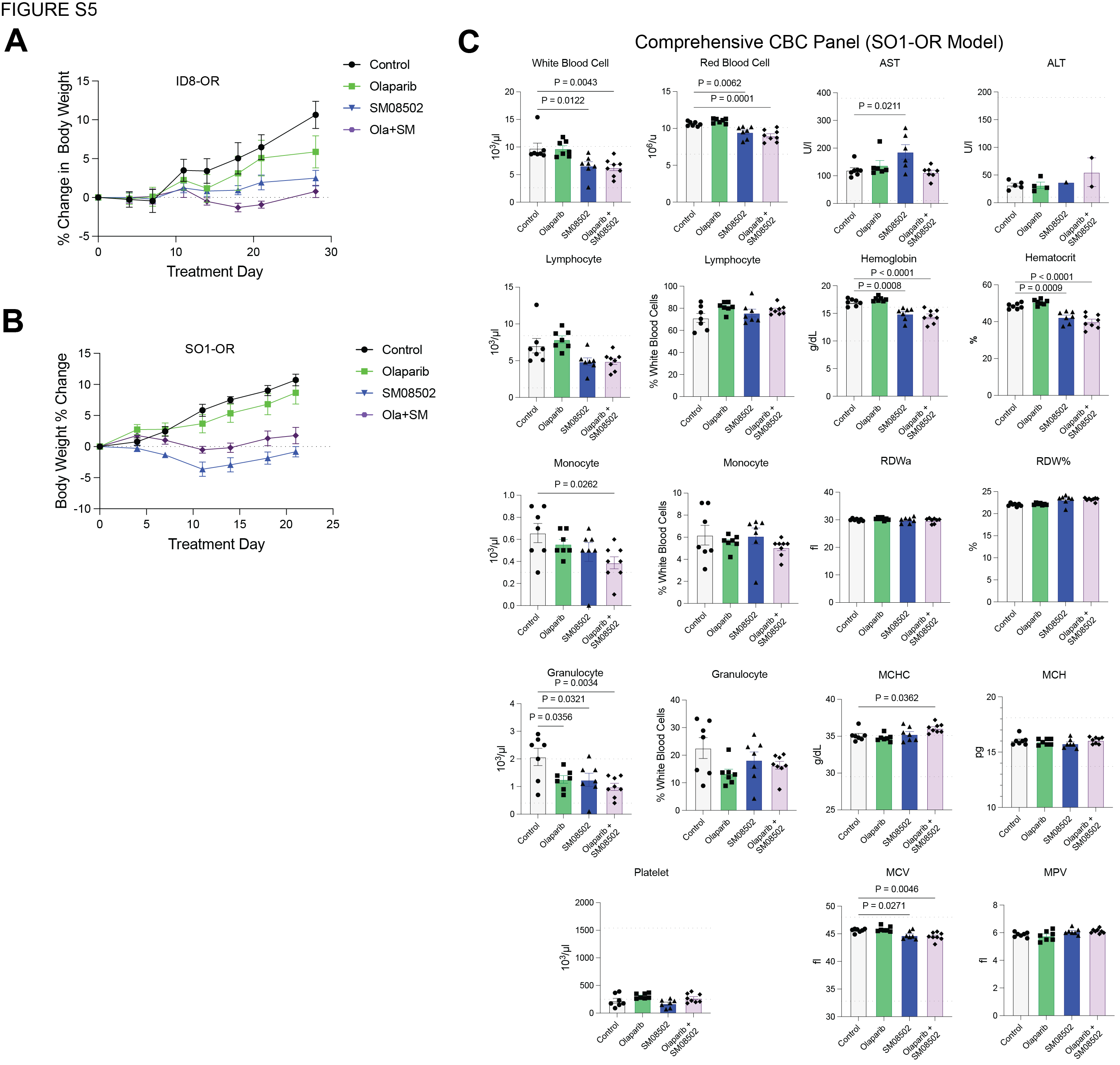
**

**Figure S5.** **Daily treatment with Olaparib and/or SM08502 does not produce overt signs of toxicity as assessed with body weight changes, CBC, or blood chemistry analysis (AST and ALT). A)** ID8-OR cells were orthotopically implanted and treated with control, olaparib, SM08502, or in combination for 28 days. Body weights are shown over time as percent change. **B)** SO1-OR cells were orthotopically implanted and treated with control, olaparib, SM08502, or in combination for 21 days. Body weights are shown over time as percent change. **C)** in the SO1-OR model - CBC and blood chemistry analysis (AST and ALT) completed on day 22. Abbreviations: CBC: complete blood count. RDW: red blood cell distribution width, MCH: mean corpuscular hemoglobin. MCHC: MCH concentration. MCV: mean corpuscular volume. Mean platelet volume. AST: aspartate aminotransferase. ALT: alanine aminotransferase. Error bars, SEM.
